# Interaction effects between sleep disorders and depression on heart failure

**DOI:** 10.1186/s12872-023-03147-5

**Published:** 2023-03-13

**Authors:** Tianshu Fan, Dechun Su

**Affiliations:** Department of Cardiology, the First Affiliated Hospital of Dalian Medical University, Liaoning Province, Dalian, 116000 China

**Keywords:** Sleep disorders, Depression, Heart failure, The National Health and Nutritional Examination Survey

## Abstract

**Background:**

Sleep disorders and depression were recognized as independent risk factors for heart failure, whether their interaction effects also correlated with the risk of heart failure remains elusive. This study was to explore the interaction effects between sleep disorders and depression on the risk of heart failure.

**Methods:**

This was a cross-sectional study that included data from 39,636 participants in the National Health and Nutritional Examination Survey (NHANES) database. Poisson regression model was applied to evaluate the associations of depression or sleep disorders with heart failure. The relative excess risk of interaction (RERI), attributable proportion of interaction (API) and synergy index (SI) were used to measure whether the interaction effects between depression and sleep disorders on heart failure was statistically significant.

**Results:**

The risk of heart failure was increased in people with sleep disorders [risk ratio (RR) = 1.92, 95% confidence interval (CI): 1.68–2.19) after adjusting for confounders including age, gender, body mass index (BMI), race, marital status, education level, annual family income, drinking history, smoking history, diabetes, hypertension and stroke. The risk of heart failure was elevated in patients with depression after adjusting for confounders (RR = 1.96, 95%CI: 1.65–2.33). Patients with depression and sleep disorders were associated with increased risk of heart failure after adjusting for confounders (RR = 2.76, 95%CI: 2.23–3.42). The CIs of interactive indexes RERI was -0.42 (95%CI: -1.23–0.39), and API was -0.15 (95%CI: -0.46–0.16), which included 0. The CI of interactive indexes SI was 0.81 (95%CI: 0.54–1.21), which contained 1.

**Conclusion:**

Depression and sleep disorders were independent risk factors for heart failure but the interaction effects between depression and sleep disorders on the occurrence of heart failure were not statistically different.

**Supplementary Information:**

The online version contains supplementary material available at 10.1186/s12872-023-03147-5.

## Background

Heart failure is a progressive and symptomatic syndrome that has been recognized as one of the main global health problems [1]. Nearly 5.7 million adults > 20 years suffered from heart failure and the estimated prevalence is about 10% in people age > 65 years [2]. Patients with heart failure require lifelong medical treatment and great health care, and this results in a high premature mortality [3]. Heart failure decreases the quality of life in patients and brings heavy burden to the society [4]. Given the high prevalence and cost of heart failure, increasing emphasis has been put on exploring the factors associated with heart failure. A better understanding of modifiable risk factors and their interaction effects on heart failure is vital for the prevention of this disease.

Sleep disorders including sleep apnea, insomnia, restless legs, and others are frequently identified in patients with heart failure [5, 6]. Almost 75% of heart failure patients were reported to have sleep disorders [7]. Sleep duration and sleep disorders were revealed to be associated with increased risk of heart failure [8, 9]. Another modifiable risk factor for heart failure might be depression in patients. Depression is a common comorbidity in heart failure and approximately 30% of heart failure patients suffer from depression and even more have depressive symptoms [10]. Multiple evidence indicated that depressive symptoms were risk factors for heart diseases [11]. Several studies also revealed that depression not only reduces the quality of life and increases the re-hospitalization rate of heart failure patients, but also increases the morbidity and mortality of heart failure and affects the prognosis of these patients [12, 13]. Growing numbers of studies showed that depression and sleep disorders had bidirectional relationship with each other [14]. Several researchers also found that the interaction effects between depression and sleep disorders affected the occurrence of stroke and type 2 diabetes [15]. At present, sleep disorders and depression were recognized as independent risk factors for heart failure, whether their interaction effects also correlated with the risk of heart failure remains elusive.

Due to the high mortality and morbidity of heart failure, an updated and careful management of different aspects that characterize the disease such as stratifying factors associated with heart failure is essential for correctly clinical managing patients [16]. Previous studies have explored the well-known clinical, laboratory and instrumental characteristics that might influence heart failure [17, 18], the demographic characteristics such as age, gender, and body mass index (BMI) also have different influences and deserve specific insights and clarifications [19–21]. At this scope, we performed the subgroup analysis to investigate the interaction effects between sleep disorders and depression on the risk of heart failure in participants with different demographic characteristics.

In the current study, we aimed to explore the interaction effects between sleep disorders and depression on the risk of heart failure based on the data from the National Health and Nutritional Examination Survey (NHANES) database. The independent as well as the bidirectional relationship between sleep disorders and depression were respectively investigated to deeply evaluate the associations of sleep disorders and depression with the risk of heart failure. We also stratified the analysis in terms of demographic characteristics such as age, gender, BMI and marital status.

## Methods

### Study design and population

This was a cross-sectional study collected the data of 39,636 participants from NHANES database. NHANES is a survey collecting the data of nationally representative samples in the United States each year and recording the detailed demographic information and comprehensive nutrition data on dietary intake, anthropometric measurements, as well as blood samples by standardized interviews and direct examination of participants [22]. In the current study, after excluding participants without the data on sleep disorders, depression questionnaires and others, 30,406 subjects were finally included.

### Data collection

The data of all subjects were collected including the age (years), gender, BMI (kg/m^2^), race (Mexican American, Hispanic, non-Hispanic White, non-Hispanic Black, or others), marital status (married, widowed, divorced/separated, or unmarried), education [Junior high and below, High school/General Equivalent Diploma (GED), Junior college or above], annual family income (< $20,000 or ≥ $20,000), drinking history, smoking history, diabetes mellitus, stroke, hypertension, sleep disorders, depression, depression severity (no, moderate, moderate to severe and severe), and heart failure. All participants were divided into the heart failure group (*n* = 977) and non-heart failure group (*n* = 29,429).

### Definitions of variables

Heart failure in patients was defined as the outcome in this study, which was determined by a response of “yes” to the household interview question asking whether they had been told to have congestive heart failure in the “Medical Conditions” module of “Questionnaire Data” in the NHANES. Sleep disorders were defined based on a response of “yes” to the question asking them whether they had been told to have sleep disorders by doctors or professional health workers. Depression was measured by the Patient Health Questionnaire 9 (PHQ-9) and PHQ-9 scores ≥ 10 was defined as depression [23]. The severity of depression was determined based on the PHQ-9 scores, including no depression (0–9), moderate depression (10–14), moderate to severe depression (15–19), and severe depression (20–27) [24]. The reliability or validity of the PHQ-9 were assessed and the Cronbach Alpha coefficient of PHQ-9 was 0.846447 after standardization.

### The additive interaction effects model

Three indexes including relative excess risk of interaction (RERI), attributable proportion of interaction (API), and synergy index (SI) were used to assess the interaction effects between sleep disorders and depression on the risk of heart failure based on the addictive model. RERI = R11-R10-R01 + 1: represents the difference between the sum of the combined effects of the two factors and the sum of the separate effects. It also represents the risk degree of interaction effects in comparison with all other factors except the two factors. API = RERI/R11: represents the proportion of total effects attributed to interaction. SI = R11 (R10 × R01): the meaning is the same as RERI. No interaction effects were shown when 0 was included in the confidence intervals (CIs) of RERI and API and 1 was involved in the CI of SI.

### Statistical analysis

The Shapiro–Wilk was applied for measuring the normality of the measurement data. The measurement data with normal distribution were described as Mean ± SD and comparisons between groups were subjected to t test. Non-normal distributed data were shown as [M (Q_1_, Q_3_)] and differences between groups were compared via Mann–Whitney U rank sum test. The enumeration data were described as n (%). Chi-square test (χ^2^) or Fisher’s exact probability method was used for comparison between the groups. Poisson regression model was applied to evaluate the associations of depression or sleep disorders with heart failure. Model 1 was the unadjusted crude model. Model 2 adjusted for demographic characteristics including age, BMI, marital status and gender. Model 3 adjusted for variables with statistical difference between heart failure group and non-heart failure group including age, gender, race, marital status, education, annual family income, drinking history, smoking history diabetes mellitus, hypertension and stroke. Stepwise regression analysis was applied in Model 3. Sensitivity analysis was performed in the data before and after deleting the missing data to explore whether the missing values influenced the results. RERI, API and SI were used to assess whether the interaction effects between depression and sleep disorders on heart failure was statistically significant. All statistical tests were performed by two-sided test and *P* < 0.05 were considered to be statistically significant. SAS 9.4 was used for statistical analysis, R 4.20 software was used to draw the forest plot, and GraphPad was applied to draw the graph showing the risk ratios (RR) of interaction effects term.

## Results

### The characteristics of all participants

**Fig. 1:**
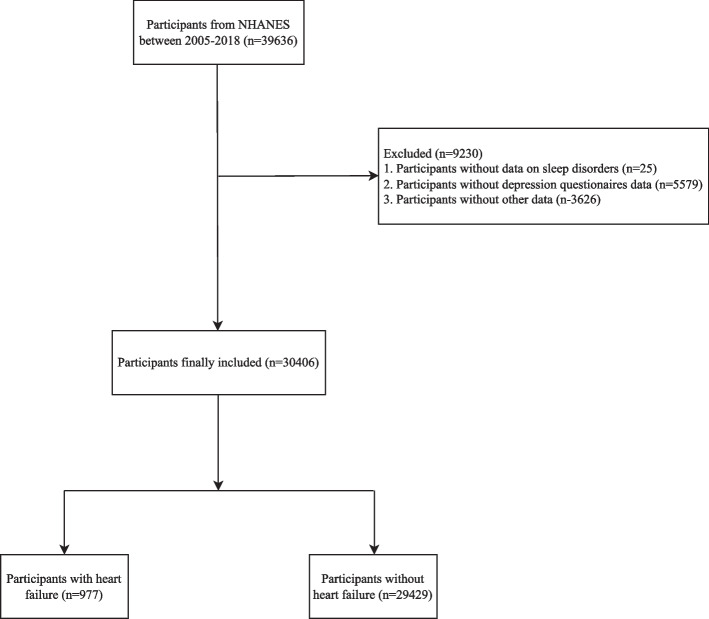
The screen process of participants in this study

The data of 39,636 participants were extracted from NHANES database. Among them, 25 people missed the data on sleep disorders, 5579 subjects had no data on depression questionnaires and 3626 persons missed other data, and 30,406 subjects were finally included. The detailed screen process was shown in Fig. 1. The median age of all the participants was 49 years. 14,998 (49.33%) of the subjects were male. The average BMI was 29.25 kg/m^2^. 4679 people were Mexican–American, accounting for 15.39%, 2748 persons were Hispanic, accounting for 9.04%, 13,503 participants were non-Hispanic White, accounting for 44.41%, 6485 people were non-Hispanic Black, accounting for 21.33%, and 2991 participants were other ethnicities, accounting for 9.84%. 7831 patients had sleep disorders, accounting for 25.75%, and 2634 people had depression, accounting for 8.66%. Among patients with depression, 1642 patients were moderate depression, accounting for 5.40%, 709 people were moderate to severe depression, accounting for 2.33%, and 283 persons were severe depression, accounting for 0.93%. 977 patients had heart failure, accounting for 3.21% (Table 1).

**Table 1:** Comparisons of the characteristics between patients with and without heart failure

Variable	Total (*n* = 30,406)	Group		Statistical magnitude	*P*
		Heart failure (*n* = 29,429)	Non-heart failure (*n* = 977)		
Age, years, M(Q_1_, Q_3_)	49 (34, 64)	48 (34, 63)	69 (60, 77)	Z = 30.471	< 0.001
Gender, n (%)				χ^2^ = 21.986	< 0.001
Male	14,998 (49.33)	14,444 (49.08)	554 (56.70)		
Female	15,408 (50.67)	14,985 (50.92)	423 (43.30)		
BMI, kg/m^2^, Mean ± SD	29.25 ± 7.00	29.15 ± 6.92	32.29 ± 8.56	t = -11.160	< 0.001
Race, n(%)				χ^2^ = 96.403	< 0.001
Mexican American	4679 (15.39)	4603 (15.64)	76 (7.78)		
Hispanic	2748 (9.04)	2678 (9.10)	70 (7.16)		
non-Hispanic White	13,503 (44.41)	12,985 (44.12)	518 (53.02)		
non-Hispanic Black	6485 (21.33)	6222 (21.14)	263 (26.92)		
others	2991 (9.84)	2941 (9.99)	50 (5.12)		
Marital status, n (%)				χ^2^ = 346.640	< 0.001
Married	15,801 (51.97)	15,337 (52.12)	464 (47.49)		
Widowed	2354 (7.74)	2142 (7.28)	212 (21.70)		
Divorced/separated	4389 (14.43)	4205 (14.29)	184 (18.83)		
Unmarried	7862 (25.86)	7745 (26.32)	117 (11.98)		
Education, n (%)				Z = -8.028	< 0.001
Junior high and below	7093 (23.33)	6788 (23.07)	305 (31.22)		
High school/GED	7076 (23.27)	6804 (23.12)	272 (27.84)		
Junior college or above	16,237 (53.40)	15,837 (53.81)	400 (40.94)		
Annual family income, $, n (%)				χ^2^ = 152.884	< 0.001
< $20,000	6482 (21.32)	6118 (20.79)	364 (37.26)		
≥ $20,000	23,924 (78.68)	23,311 (79.21)	613 (62.74)		
Drinking history, n (%)	20,952 (68.91)	20,351 (69.15)	601 (61.51)	χ^2^ = 25.748	< 0.001
Smoking history, n (%)	13,882 (45.66)	13,273 (45.10)	609 (62.33)	χ^2^ = 113.169	< 0.001
Diabetes mellitus, n (%)	4021 (13.22)	3591 (12.20)	430 (44.01)	χ^2^ = 833.809	< 0.001
Stroke, n (%)	1127 (3.71)	930 (3.16)	197 (20.16)	χ^2^ = 766.006	< 0.001
Hypertension, n (%)	14,073 (46.28)	13,337 (45.32)	736 (75.33)	χ^2^ = 342.6158	< 0.0001
Sleep disorders, n (%)	7831 (25.75)	7357 (25.00)	474 (48.52)	χ^2^ = 273.488	< 0.001
Depression, n (%)	2634 (8.66)	2451 (8.33)	183 (18.73)	χ^2^ = 129.320	< 0.001
Depression severity, n (%)				Z = 11.450	< 0.001
No	27,772 (91.34)	26,978 (91.67)	794 (81.27)		
Moderate	1642 (5.40)	1537 (5.22)	105 (10.75)		
Moderate to severe	709 (2.33)	653 (2.22)	56 (5.73)		
Severe	283 (0.93)	261 (0.89)	22 (2.25)		

*BMI* Body mass index, *GED *General equivalent diploma

### Comparisons of the characteristics between patients with and without heart failure

The median age (69 years vs 48 years, Z = 30.471, *P* < 0.001), and median BMI (32.29 kg/m^2^ vs 29.15 kg/m^2^, t = 11.160, *P* < 0.001) of participants with heart failure were higher than those without. The proportions of males (56.70% vs 49.08%, χ^2^ = 21.986, *P* < 0.001), subjects with annual family income < $20,000 (χ^2^ = 152.884, *P* < 0.001), patients with smoking history (62.33% vs 45.10%, χ^2^ = 113.169, *P* < 0.001), patients with diabetes (χ^2^ = 833.809, *P* < 0.001), patients with stroke (20.16% vs 3.16%, χ^2^ = 766.006, *P* < 0.001), patients with sleep disorders (48.52% vs 25%, χ^2^ = 273.488, *P* < 0.001), patients with depression (18.73% vs 8.33%, χ^2^ = 129.320, *P* < 0.001) and different depression degrees (Z = 11.450, *P* < 0.001) in the heart failure group were higher than the non-heart failure group. The percentages of people with different education levels (Z = -8.028, *P* < 0.001) and drinking history (61.51% vs 69.15% χ^2^ = 25.748, *P* < 0.001) in people with heart failure were lower than those without heart failure. The differences concerning race (χ^2^ = 96.403, *P* < 0.001) and marital status (χ^2^ = 346.640, *P* < 0.001) between people with and without heart failure were statistically significant (Table 1).

### Associations of sleep disorders or depression with heart failure

**Fig. 2:**
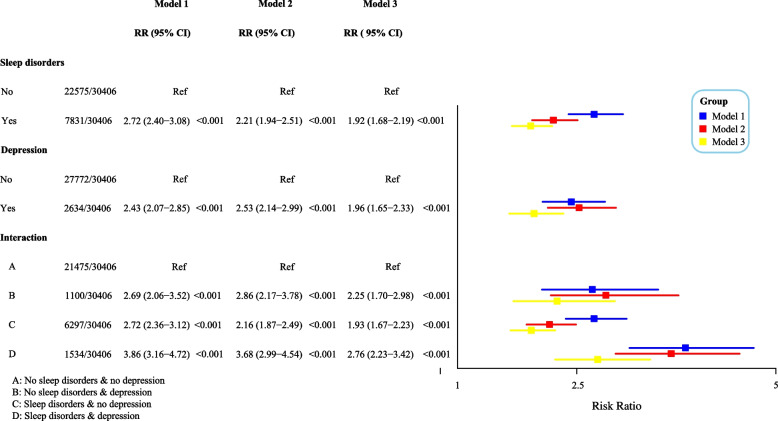
Forest plot showing the association between sleep disorders or/and depression and heart failure

**Fig. 3:**
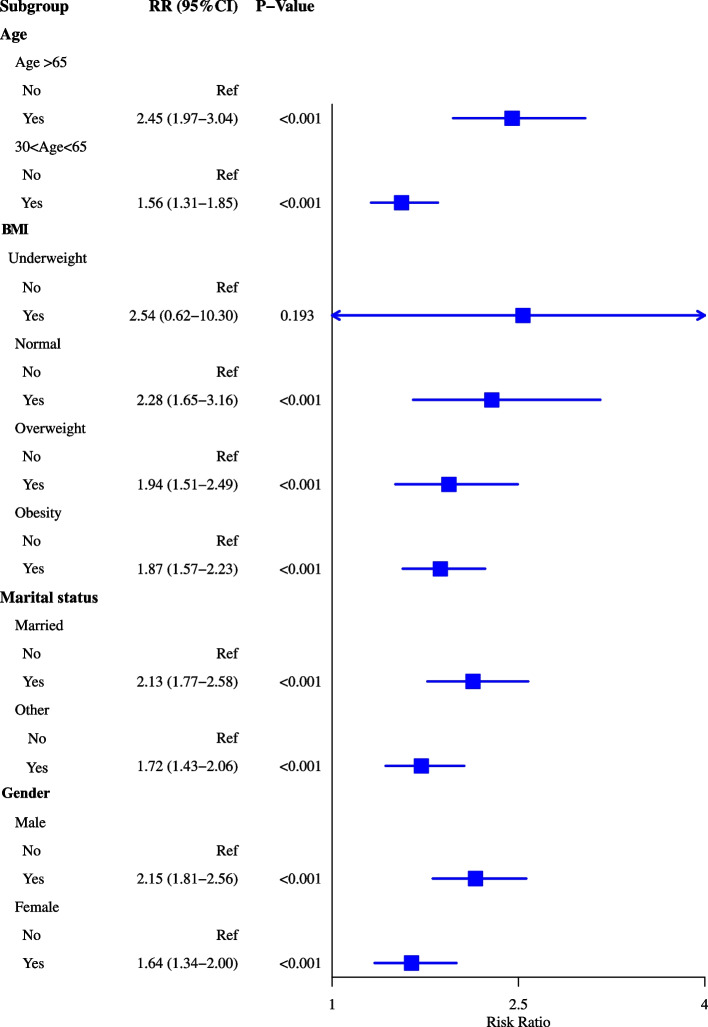
Forest plot showing the association between sleep disorders and heart failure in people with different demographic characteristics

**Fig. 4:**
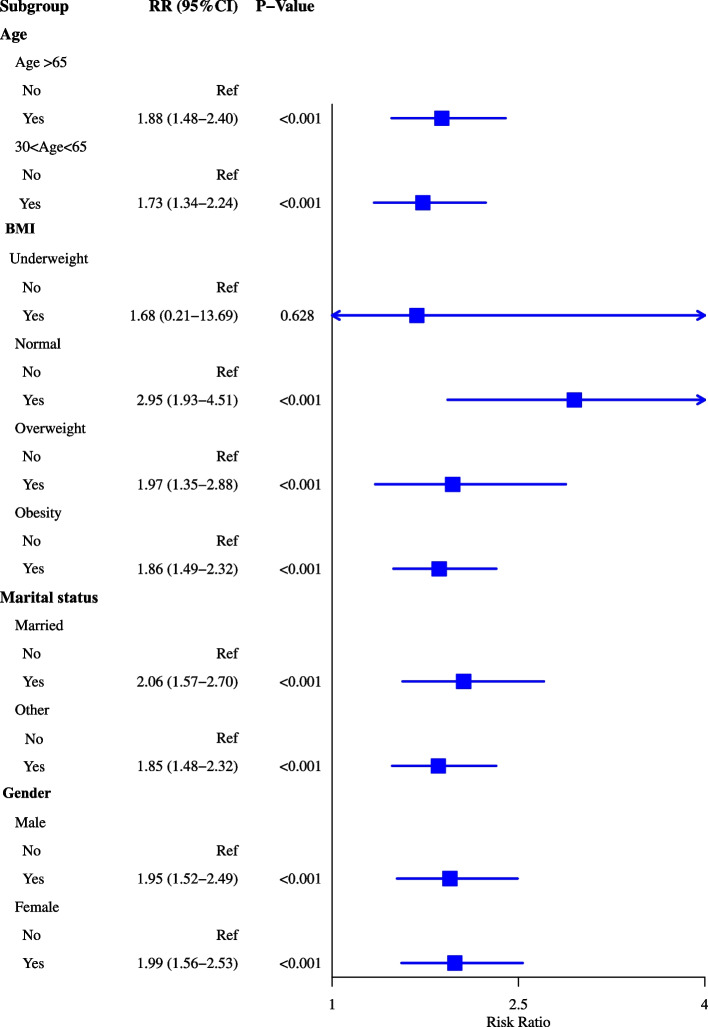
Forest plot showing the association between depression and heart failure in people with different demographic characteristics

As observed in Fig. 2, the risk of heart failure was 2.21 times increase in patients with sleep disorders in the adjusted model for age and sex (RR = 2.21, 95%CI: 1.94–2.51). After adjusting for age, sex, BMI, race, marital status, education level, annual family income, drinking history, smoking history, diabetes, hypertension and stroke, the risk of heart failure was 1.92-fold increase in people with sleep disorders (RR = 1.92, 95%CI: 1.68–2.19). The risk of heart failure was 2.53-fold increase in patients with depression after adjusting for age and sex (RR = 2.53, 95%CI: 2.14–2.99). The risk of heart failure was 1.96-fold increase in patients with depression compared with those without (RR = 1.96, 95%CI: 1.65–2.33) after adjusting for age, sex, BMI, race, marital status, education level, annual family income, drinking history, smoking history, diabetes, hypertension and stroke (Fig. 2). For most categories of age, BMI, marital status and gender, the risk ratios for sleep disorders and depression were statistically significantly (greater than 1.0) in most cases. Exception was in underweight group. The detailed information of the subgroups was exhibited in Figs. 3 and 4.

### Interaction effects between sleep disorders and depression on heart failure

**Fig. 5:**
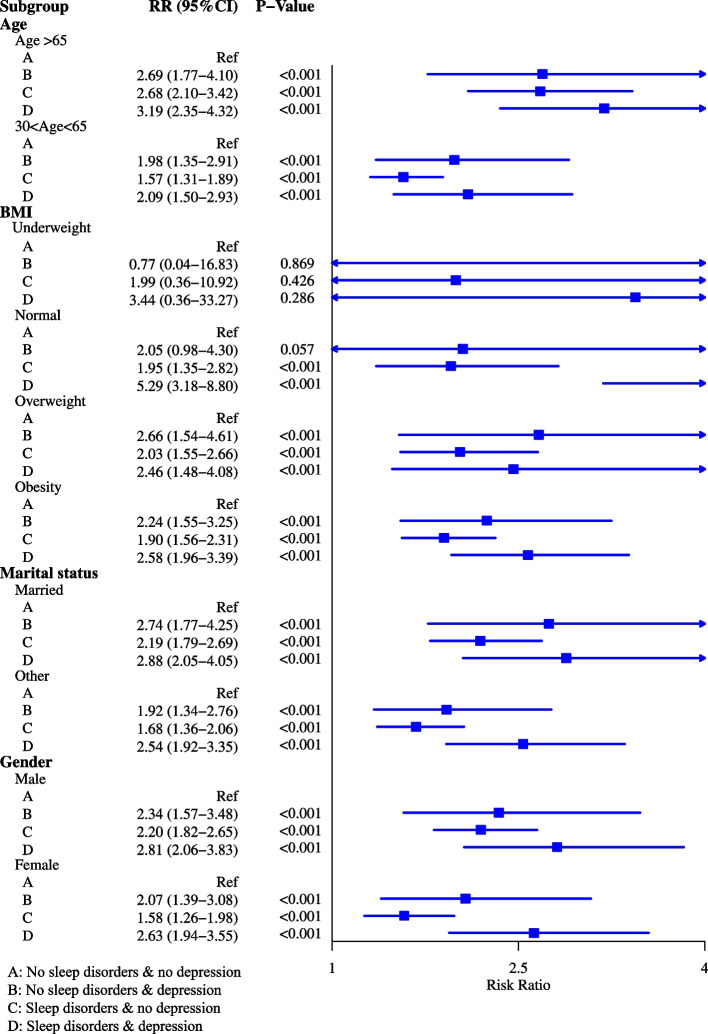
Forest plot showing the association of sleep disorders and depression with heart failure in people with different demographic characteristics

**Fig. 6:**
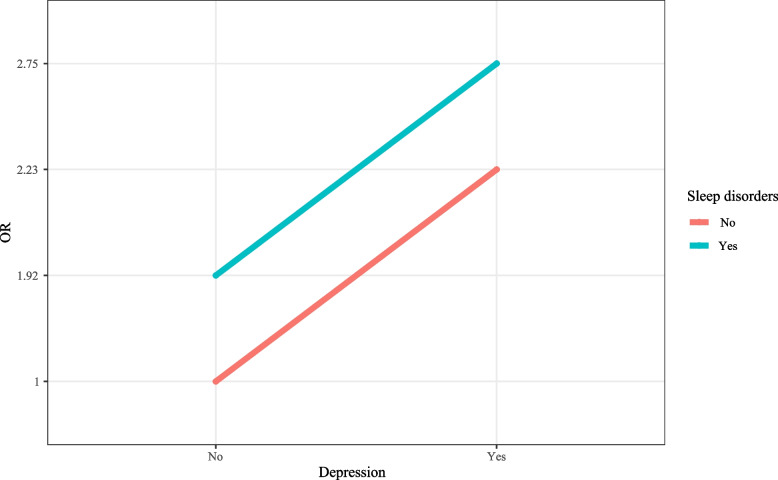
Interaction effects between sleep disorders and depression on heart failure after adjusting for confounders

The additive interaction effects terms of sleep disorders and depression were established, including no depression and no sleep disorders, no depression and sleep disorders, depression and no sleep disorders, depression and sleep disorders. The detailed sample size of each interaction effects term was displayed in Table 2. Patients with depression and sleep disorders were associated with increased risk of heart failure after adjusting for age and gender (RR = 3.68, 95% CI: 2.99–4.54), or adjusting for age, gender, BMI, race, marital status, education level, annual family income, drinking history, smoking history, diabetes, hypertension and stroke (RR = 2.76, 95%CI: 2.23–3.42) (Fig. 2). For most categories of age, BMI, marital status and gender, the risk ratios for people with sleep disorders and depression were statistically significantly (> 1.0) in most cases (Fig. 5). Sensitivity analysis depicted that there was no statistical difference of the results between the data before and after deleting the missing values (Supplementary Table 1). The CIs of interactive indexes RERI was -0.42 (95%CI: -1.23–0.39), and API was -0.15 (95%CI: -0.46–0.16), which included 0. The CIs of interactive indexes SI was 0.81 (95%CI: 0.54–1.21), which contained 1 (Table 3). These indicated that the interaction effects between sleep disorders and depression on heart failure was not statistically significant (Fig. 6). Subgroup analysis concerning the demographic characteristics exhibited no statistical differences in terms of the interaction effects between depression and sleep disorders on the risk of heart failure (Table 3).

**Table 2:** The detailed sample size of each interaction effects term

Heart failure	Depression		Sleep disorder	OR	
	Yes	No		Depression (Yes)	Depression (No)
Yes	122	352	Yes	R11	R10
No	1412	5945			
Yes	61	442	No	R01	R00
No	1039	21,033

**Table 3:** Interaction effects between sleep disorders and depression on heart failure

	RERI (CI: 95%)	API (CI: 95%)	SI (CI: 95%)
Total	-0.42 (-1.23, 0.39)	-0.15 (-0.46, 0.16)	0.81 (0.54, 1.21)
Age			
> 65	-1.18 (-2.54, 0.18)	-0.37 (-0.84, 0.10)	0.65 (0.41, 1.04)
30–65	-0.46 (-1.47, 0.54)	-0.22 (-0.74, 0.30)	0.70 (0.33, 1.51)
BMI			
Underweight	1.68 (-5.53, 8.88)	0.49 (-0.86, 1.83)	3.19 (0.01, 721.80)
Normal	2.29 (-0.54, 5.11)	0.43 (0.05, 0.82)	2.14 (0.83, 5.55)
Overweight	-1.23 (-3.09, 0.63)	-0.50 (-1.41, 0.41)	0.54 (0.21, 1.40)
Obesity	-0.57 (-1.59, 0.45)	-0.22 (-0.64, 0.20)	0.74 (0.43, 1.25)
Marital status			
Married	-1.05 (-2.53, 0.42)	-0.36 (-0.94, 0.21)	0.64 (0.35, 1.18)
Others	-0.06 (-0.98, 0.86)	-0.02 (-0.39, 0.34)	0.96 (0.54, 1.73)
Gender			
Male	-0.73 (-1.95, 0.49)	-0.26 (-0.74, 0.22)	0.71 (0.41, 1.25)
Female	-0.03 (-1.06, 1.01)	-0.01 (-0.41, 0.39)	0.98 (0.52, 1.85)

*CI* Confidence interval, *BMI* Body weight index, *RERI* Relative excess risk, *API* Attributable proportion of interaction, *SI* Synergy index

## Discussion

In the present study, 30,406 eligible participants were enrolled from NHANES, including 977 people with heart failure and 29,429 without heart failure. The results depicted that depression and sleep disorders were independently associated with increased risk of heart failure. No synergic and additive interaction effects between depression and sleep disorders on the occurrence of heart failure was obtained. The findings of this study might make it more clear about the effects between depression and sleep disorders on the occurrence of heart failure, and help the clinicians to make appropriate interventions on patients with depression or/and sleep disorders.

Depression is a chronic medical illness affecting thoughts, mood, and physical health, which decreases the ability of individuals to function in their daily life [25]. A review by Celano et al. uncovered that depression was associated with the development and progression of heart failure via mediating the physiologic and behavioral mechanisms [26]. Another prospective observational study including about 2 million healthy adults demonstrated that depression was prospectively associated with an 18% increased risk of heart failure [27]. These findings gave support to the results of our study, which showed that depression was a risk factor for the occurrence of heart failure. This may be because patients with depression was linked to the hypothalamic–pituitary–adrenal gland dysfunction, increased pro-inflammatory and pro-thrombotic factor activity, reduced heart rate variability and physical inactivity [28]. Depressive symptoms and major depression were associated with elevated levels of inflammatory biomarkers such as C-reactive protein (CRP), interleukin (IL)-1, IL-6, tumor necrosis factor-alpha (TNF-α) and monocyte chemoattractant protein-1 (MCP-1) [29]. The immune system and inflammation were reported to be involved in the pathogenesis of heart failure [30]. To early screen out patients with depression can timely provide proper treatments such as anti-depressive medications. In the current study, sleep disorders were recognized to be associated with a higher risk of heart failure. Javaheri et al. discovered that insomnia, especially when accompanied by short sleep duration was linked with increased risk of heart failure [31]. A prospective population-based study reported that obstructive sleep apnea had a twofold increase in the risk of heart failure [32]. The potential mechanisms might be that sleep disorders including sleep-disordered breathing is associated with increased sympathetic activation, vagal withdrawal, altered haemodynamic loading conditions, and hypoxaemia, which is one of the most common risk factor for cardiac failure [33]. To improve the quality of sleep in general population might be a strategy for the prevention of heart failure. Subgroup analysis showed that depression and sleep disorders were associated with higher risk of heart failure in both patients aged ≥ 65 years and 30–65 years, married or others, males or females. These suggested that patients with sleep disorders or depression should be cautious of the risk of heart failure despite the age, marital status and gender. As for underweight patients, no significant association was found between depression or sleep disorders and heart failure. This maybe because the sample size in underweight group was small [underweight group (*n* = 475) vs normal BMI (*n* = 8521) vs overweight (*n* = 9945) vs obesity (*n* = 11,465)].

In this study, the interaction effects between depression and sleep disorders on heart failure were also explored, and no synergic interaction effects between depression and sleep disorders was identified on the occurrence of heart failure. This maybe because the disease course of heart failure was progressive and long [34], and the interaction effects of depression and sleep disorders on heart failure was not significant during the long disease course. The interaction effects between depression and sleep disorders on heart failure might be more obvious if more relevant clinical biomarkers were including in the subgroup analysis. Inflammation plays an important part in depression, sleep disorders and heart failure [35, 36], and depression and sleep disorders might be linked to heart failure through the pathway of inflammation. Depression and heart failure also share some mechanisms and risk factors, including dysregulation of platelet reactivity, neuroendocrine function, arrhythmias, high-risk behaviors, and social factors [10]. Severe depression was reported to be associated with diastolic dysfunction and left ventricular hypertrophy, which increase the risk of heart failure [37]. For patients with depression, early interventions should be provided to decrease the risk of heart failure in those patients. Interestingly, we found in people with normal BMI, there might be interaction effects of depression and sleep disorders on heart failure. This maybe because abnormal BMI might involve in more mechanisms associated with the occurrence of heart failure [38], and the interaction effects of depression and sleep disorders on heart failure might be not significant.

This study explored the interaction effects between depression and sleep disorders on heart failure, which obtained convincing results based on the nationally representative NHANES database with a large sample size. Several limitations existed in our study. Firstly, this was a cross-sectional study, which could only identify the associations but not the causal relationship between depression or sleep disorders and heart failure. Secondly, the history of sleep disorders and heart failure were based on the self-reported data of participants in the NHANES, which might cause bias. Thirdly, subgroup analysis was only conducted in terms of gender, race and age, more heart failure related subgroups should be performed to clearly identify the interaction effects between depression and sleep disorders on heart failure in different populations. In the future, more case–control studies on deeply exploring the interaction effects of depression and sleep disorders on heart failure were required to verify the findings in the current study.

## Conclusions

Our study analyzed the effect of depression and sleep disorders on the risk of heart failure based on the data of 30,406 participants from NHANES. The findings revealed that depression and sleep disorders were independent risk factors of heart failure but the interaction effects between depression and sleep disorders were not statistically different on the occurrence of heart failure. The results of our study revealed the co-existing of sleep disorder and depression does not seem to have a synergistic effect on the occurrence of heart failure.

## Supplementary Information

**Additional file 1: Table1. **Comparisons of the results between the data before and after deleting themissing values.

## Data Availability

The datasets generated and/or analyzed during the current study are available in the NHANES repository, https://www.cdc.gov/nchs/nhanes/.

## Abbreviations

NHANES: National Health and Nutritional Examination Survey
GED: General Equivalent Diploma
PHQ-9: Patient Health Questionnaire 9
RERI: Relative excess risk of interaction
API: Attributable proportion of interaction
SI: Synergy index
CIs: Confidence intervals
