# Supplementary material for: Interaction effects between sleep disorders and depression on heart failure
Source: BMC Cardiovasc Disord. 2023 Mar 13;23:132. doi: 10.1186/s12872-023-03147-5 (PMC10009973; doi:10.1186/s12872-023-03147-5)
Supplement: Supplementary file 1 — Additional file 1: Table1. Comparisons of the results between the data before and after deleting themissing values. [file 12872_2023_3147_MOESM1_ESM.docx]

Supplementary Table 1 Comparisons of the results between the data before and after deleting the missing values

| Variables | Model 1 | | Model 2 | | Model 3 | |
| --- | --- | --- | --- | --- | --- | --- |
|  | RR (95%CI) | *P* | RR (95%CI) | *P* | RR (95%CI) | *P* |
| Data after deleting the missing values | | | | | | |
| Sleep disorders |  |  |  |  |  |  |
| No |  |  | Ref |  | Ref |  |
| Yes | 2.72 (2.40-3.08) | <.001 | 2.21 (1.94-2.51) | <.001 | 1.92 (1.68-2.19) | <.001 |
| Depression |  |  |  |  |  |  |
| No | Ref |  | Ref |  | Ref |  |
| Yes | 2.43 (2.07-2.85) | <.001 | 2.53 (2.14-2.99) | <.001 | 1.95 (1.64-2.31) | <.001 |
| Interaction effects |  |  |  |  |  |  |
| A | Ref |  | Ref |  | Ref |  |
| B | 2.69 (2.06-3.52) | <.001 | 2.86 (2.17-3.78) | <.001 | 2.22 (1.68-2.93) | <.001 |
| C | 2.72 (2.36-3.12) | <.001 | 2.16 (1.87-2.49) | <.001 | 1.92 (1.66-2.22) | <.001 |
| D | 3.86 (3.16-4.72) | <.001 | 3.68 (2.99-4.54) | <.001 | 2.74 (2.21-3.40) | <.001 |
| Data before deleting the missing values | | | | | | |
| Sleep disorders |  |  |  |  |  |  |
| No | Ref |  | Ref |  | Ref |  |
| Yes | 2.73 (2.45-3.04) | <.001 | 2.26 (2.01-2.53) | <.001 | 1.94 (1.72-2.19) | <.001 |
| Depression |  |  |  |  |  |  |
| No | Ref |  | Ref |  | Ref |  |
| Yes | 2.45 (2.11-2.83) | <.001 | 2.39 (2.06-2.79) | <.001 | 1.89 (1.61-2.21) | <.001 |
| Interaction effects |  |  |  |  |  |  |
| A | Ref |  | Ref |  | Ref |  |
| B | 2.59 (2.03-3.31) | <.001 | 2.51 (1.95-3.22) | <.001 | 2.05 (1.58-2.64) | <.001 |
| C | 2.74 (2.41-3.13) | <.001 | 2.20 (1.92-2.52) | <.001 | 1.95 (1.70-2.24) | <.001 |
| D | 4.04 (3.37-4.84) | <.001 | 3.73 (3.08-4.51) | <.001 | 2.82 (2.31-3.43) | <.001 |

A: No sleep disorders & no depression

B: No sleep disorders & depression

C: Sleep disorders & no depression

D: Sleep disorders & depression

RR: risk ratio
